# Correlates of condom use among school-going Thai adolescents: the critical role of bullying victimizations

**DOI:** 10.1186/s12888-024-06423-6

**Published:** 2025-01-03

**Authors:** Omid Dadras

**Affiliations:** 1 Department of Global Public Health and Primary Care, University of Bergen, Årstadveien 17, Bergen, 5009 Norway; 2 Research Centre for Child Psychiatry, University of Turku, Turku, Finland

**Keywords:** Bullying, Condom use, Thailand, Adolescents, Correlates

## Abstract

**Background:**

Adolescents face numerous challenges that influence their sexual behaviors. Among these, bullying victimization is a critical yet understudied factor that may impact engagement in unprotected sex. This study investigated the correlates of condom use among school-going Thai adolescents, with a main focus on bullying victimization.

**Methods:**

This study is a secondary analysis of the Global School-based Student Health Survey (GSHS) conducted in 2021 among a nationally representative sample of students in grades 7–12 in Thailand. The analysis included all the sexually active students with condom use in the last sex as the main outcome variable. Bivariate binary logistic regression analysis was used to examine the odds of using condom across various explanatory variables. Additional adjusted logistic regression models were constructed to examine the association between bullying experiences and condom use while controlling for the potential confounding effect of other explanatory variables.

**Results:**

Among sexually active participants, 416 (69%) reported using condoms in their last sex and more than half of them reported bullying experiences. Besides bullying, this study identifies several factors such as older age (< 14) and higher grades that were associated with lower use of condoms. Additionally, other psychosocial factors such as suicidal ideation and attempts as well as physical violence, and lack of close friends decrease the odds of condom use. Substance use, particularly marijuana and amphetamine/methamphetamine, was also associated with lower condom use. Although all types of bullying experiences were associated with lower condom use in bivariate analyses, the association remained significant only for bullying at school in the adjusted model (AOR = 0.39, 95% CI: 0.18–0.86).

**Conclusion:**

The findings emphasize a holistic approach to addressing bullying, particularly within schools, and promoting protected sexual behaviors among school-going adolescents through early anti-bullying interventions and incorporating tailored sex education into school curricula, and at Thai schools.

## Introduction

Adolescents face numerous challenges that influence their sexual behaviors and experiences. Among these, bullying victimization has emerged as a critical yet understudied factor that may significantly impact adolescents’ engagement in unprotected sexual practices [1, 2]. Bullying is a prevalent issue among adolescents worldwide, with detrimental effects on the mental, emotional, and physical health of adolescents [3–5]. Research has consistently shown a link between bullying and engagement in risky sexual behaviors among adolescents [6–8]. Both bullies and bully victims are more likely to engage in casual sex and sex under the influence of alcohol or drugs [6]. Moreover, bullying is associated with HIV-risk behaviors among young men who have sex with men [7]. Additionally, bullied students reported higher levels of engagement in various risk behaviors, including substance use and self-harm [8].

In Thailand, the prevalence of bullying is high, with approximately a fifth of school-going adolescents reporting being bullied in 2021 [9]. This can have profound effects on their behaviors and attitudes, including health-related behaviors such as condom use [3]. Additionally, cyberbullying is a growing concern among the Thai population, with adolescents being both perpetrators and victims [10]. While existing research has predominantly focused on identifying correlates of bullying and its implications on self-reported mental health among Thai adolescents [11, 12], there is a notable gap in exploring the intricate relationship between bullying incidents and risky sexual behaviors such as condom use.

Given these gaps in the literature, this study aims to examine the associations between various forms of bullying victimization (at school, outside school, and cyberbullying) and condom use among Thai adolescents, both in unadjusted and adjusted analyses. Specifically, we hypothesize that bullying victimization can be associated with a reduced likelihood of condom use during the last sexual encounter among Thai adolescents. The rationale behind this study lies in the potential implications for developing targeted interventions that address both bullying prevention and promotion of condom use among Thai adolescents, ultimately improving their overall well-being and reducing their risk of negative sexual health outcomes.

## Methods

### Survey setting

The Thailand GSHS 2021 is a nationally representative survey of school-based participants in grades 7–12, typically targeting adolescents aged 13–17 years. The survey encompassed several areas, including alcohol use, dietary habits, drug use, hygiene, mental health, physical activity, protective factors, sexual behaviors, tobacco use, and experiences of violence and unintentional injury.

### Sampling and response rate

After informed consent, students were approached in the classroom and provided with the questionnaire and recorded their responses on a computer-scannable answer sheet. The GSHS 2021 employed a two-phase cluster sampling approach to ensure the comprehensive representation of students in grades 7–12 across Thailand. Initially, schools were chosen based on their enrollment size using a probability proportional to their size. Subsequently, classes were randomly selected, and all students within those classes were eligible to participate. The survey achieved a school response rate of 92% and a student response rate of 90%, culminating in an overall response rate of 83%. A total of 5,661 students took part in the Thailand GSHS 2021 survey.

### Study variables

#### Outcome variable

Condom use in the last sex was considered as the protected sex. It is defined by asking the question “The last time you had sexual intercourse, did you or your partner use a condom?” with the alternative responses of “yes” or “no”.

#### Explanatory variables

The explanatory variables were selected based on their relevancy and effects on bullying and condom use as well as the availability of the data from Thailand GSHS 2021. These include demographic variables including age, sex, and grade; psychosocial harms and support-related variables including felt lonely and anxiety-induced insomnia in the past year (rarely/never, most of time/always), suicide ideation/attempt or being physically attacked/engage in a physical fight in the past year (yes, no), missed school/classes without permission in the past month, parent understand their problem/know about the free time (rarely/never, most of time/always), have a close friend (yes, no); and lastly substance use related variables including currently use marijuana/cigarette (in the past month), ever amphetamine use (yes, no), and tried cigarette/alcohol/drugs before age 14 (yes, no).

Traditional bullying was assessed through inquiries such as " During the past 12 months, have you ever been bullied on school property?” and “Have you encountered bullying outside of school grounds in the last year?” with response options of “yes” (1) or “no” (0). Cyberbullying was evaluated by asking, " During the past 12 months, have you ever been cyberbullied? (Count being bullied through texting, Instagram, Snapchat, Facebook, Twitter, TikTok, or other social media.). Responses to this question were also binary, with options of “yes” (1) or “no” (0).

### Statistical analysis

The descriptive statistics described the distribution of demographic characteristics, psychosocial and substance use-related variables, those who ever-had sex, and those who used a condom in their last sex among the study sample. Bivariate binary logistic regression analysis was used to examine the odds of using condom across various explanatory variables. Subsequently, additional adjusted logistic regression models were constructed, incorporating the significant variables identified in bivariate analysis (*p* < 0.05), to examine the association between bullying experiences and condom use while controlling for the potential confounding effect of other explanatory variables. The optimal model was determined using a stepwise backward approach, retaining covariates that provided the best fit. The likelihood ratio test was employed to select the most suitable model among nested models. Multicollinearity was assessed using the “collin” command in STATA to ensure model integrity. Results were presented as odds ratios (OR) with corresponding 95% confidence intervals (95% CI). To address the survey design, appropriate adjustments such as defining survey strata, primary sampling unit, and weight were applied using STATA 17. Statistical significance was defined as *p* < 0.05.

## Results

A total of 5358 students in grades 7-12th participated in Thailand GSHS 2021. Of those, 902 reported ever having sex. Among sexually active participants, 416 students reported using a condom in their last sex.

### The association of psychosocial factors and substance use with condom use

Adolescents aged over 14 were more likely to have had sex compared to those aged 14 or younger (24.44% vs. 13.39%, respectively). Among those who had sex, older adolescents (> 14) were significantly more likely to have used a condom in their last sexual encounter compared to younger ones (OR = 2.09, 95% CI: 1.43–3.03). Males were more likely to report having had sex compared to females (23.77% vs. 16.65%, respectively). However, among sexually active participants, there were no significant differences in condom use between the opposite sex (*p* > 0.05). The likelihood of using a condom increased with higher grades. students in the 12th grade had the highest rate of condom use (76.56%), followed by 11th graders (76.78%). The odds of using a condom were significantly higher for students in the 9th, 10th, 11th, and 12th grades compared to those in the 7th grade (Table 1).

**Table 1:** The demographic characteristics and condom use in last sex among Thai adolescents in grades 7-12th

	Ever had sex	Used condom in last sex	
	N (%)	N (%)	OR (95%CI)
Total	902 (19.8)	416 (69.0)	-
Age group			
≤ 14	275 (13.39)	70 (55.85)	Ref
> 14	627 (24.44)	346 (72.51)	2.09 (1.43, 3.03)**
Sex			
Male	462 (23.77)	221 (70.77)	Ref
Female	439 (16.65)	195 (66.86)	0.83 (0.54, 1.28)
Grade			
7^th^	156 (10.76)	28 (44.43)	Ref
8^th^	126 (17.74)	44 (57.01)	1.66 (0.63, 4.33)
9^th^	193 (16.18)	91 (67.09)	2.55 (1.18, 5.49)*
10^th^	75 (18.07)	36 (73.34)	3.44 (1.11, 10.63)*
11^th^	121 (31.71)	63 (76.78)	4.14 (1.74, 9.84)*
12^th^	227 (38.45)	152 (76.56)	4.09 (1.71, 9.78)*

* p-value < 0.05. **p-value < 0.001

There was no significant difference in the odds of engaging in sex among adolescents who reported feeling lonely and experiencing anxiety-induced insomnia as compared to those who did not (Table 2). Adolescents reporting suicide ideation, suicide attempts, being physically attacked, or engaging in physical fights were significantly less likely to have used condoms compared to their counterparts, as indicated by odds ratios below 1 and statistically significant p-values (*p* < 0.05, Table 2). Adolescents who reported having no close friends (28.04%) were significantly less likely to have used condoms (compared to those who reported having close friends (52.33% vs. 19.24%, respectively; OR = 0.46), indicating a potential protective effect of social support on condom use.

**Table 2:** The psychosocial distress and support and condom use in last sex among Thai adolescents in grades 7-12^th^

	Ever had sex	Used condom in last sex	
	N (%)	N (%)	OR (95%CI)
Felt lonely			
No	694 (18.35)	311 (69.89)	Ref
Yes	196 (25.29)	99 (66.74)	0.86 (0.48, 1.54)
Anxiety-induced insomnia			
No	701 (18.37)	324 (70.76)	
Yes	189 (26.67)	87 (65.33)	0.78 (0.49, 1.23)
Suicide ideation			
No	676 (17.97)	317 (73.37)	Ref
Yes	184 (26.10)	79 (58.21)	0.51 (0.33, 0.78)*
Suicide attempt			
No	697 (17.92)	329 (73.02)	Ref
Yes	197 (31.10)	84 (57.95)	0.51 (0.29, 0.88)*
Being physically attacked			
No	664 (18.09)	327 (73.64)	Ref
Yes	221 (27.66)	82 (54.31)	0.43 (0.25, 0.73)*
Engage in a physical fight			
No	604 (17.28)	282 (74.39)	Ref
Yes	297 (28.74)	134 (59.62)	0.51 (0.28, 0.93)*
Missed classes/school without permission			
No	583 (16.01)	262 (72.10)	Ref
Yes	306 (37.01)	147 (62.97)	0.65 (0.37, 1.12)
Parent understand problem			
No	673 (20.94)	294 (67.96)	Ref
Yes	202 (15.91)	112 (76.87)	1.57 (0.93, 2.63)
Have no close friend			
No	809 (19.24)	384 (70.37)	Ref
Yes	81 (28.04)	28 (52.33)	0.46 (0.24, 0.91)*

* p-value < 0.05. **p-value < 0.001

Adolescents currently using marijuana were significantly less likely to use condoms during their last sexual encounter compared to non-users (OR = 0.36, 95% CI: 0.21–0.62). Similarly, adolescents who had ever used amphetamine/methamphetamine were less likely to use condoms compared to non-users (OR = 0.47, 95% CI: 0.27–0.83). Although not statistically significant, there was a trend suggesting that current cigarette smokers were less likely to use condoms during their last sexual encounter compared to non-smokers (OR = 0.78, 95% CI: 0.47–1.30). No significant association was found between current alcohol use and condom use during the last sexual encounter (Table 3). Initiating alcohol consumption and cigarette smoking before the age of 14 was associated with decreased odds of condom use during the last sexual encounter (OR = 0.53, 95% CI: 0.32–0.89 and OR = 0.38, 95% CI: 0.20–0.76, respectively). However, there was no significant association between initiating drug use before the age of 14 and condom use during the last sexual encounter (Table 3).

**Table 3:** Substance use and condom use in last sex among Thai adolescents in grades 7-12th

	Ever had sex	Used condom in last sex	
	N (%)	N (%)	OR (95%CI)
Currently use marijuana			
No	763 (17.90)	349 (73.37)	Ref
Yes	84 (54.04)	39 (49.40)	0.36 (0.21, 0.62)**
Ever used amphetamine/methamphetamine			
No	781 (18.11)	361 (71.05)	Ref
Yes	75 (66.28)	40 (53.78)	0.47 (0.27, 0.83)*
Currently use cigarette			
No	661 (16.63)	293 (71.11)	Ref
Yes	207 (46.00)	112 (65.86)	0.78 (0.47, 1.30)
Currently use alcohol			
No	467 (13.48)	168 (71.23)	Ref
Yes	407 (35.55)	238 (68.31)	0.87 (0.62, 1.22)
Drank alcohol before age 14			
No	310 (28.77)	197 (75.57)	Ref
Yes	307 (28.53)	151 (62.26)	0.53 (0.32, 0.89)*
Tried a cigarette before age 14			
No	155 (48.28)	106 (79.60)	Ref
Yes	248 (29.66)	116 (59.99)	0.38 (0.20, 0.76)*
Used drugs before age 14			
No	67 (52.14)	45 (71.02)	Ref
Yes	129 (43.36)	45 (71.02)	0.41 (0.11, 1.49)

* p-value < 0.05. **p-value < 0.001

**Table 4:** Association of traditional (at/outside school) and cyberbullying with condom use in last sex among Thai adolescents in grades 7-12th

	Ever had sex	Used condom in last sex		
	N (%)	N (%)	OR (95%CI)	AOR (95%CI) ^a^
Bullied at school				
No	669 (19.1)	340 (74.2)	Ref	Ref
Yes	199 (20.6)	61(49.9)	0.35 (0.19, 0.64)*	0.39 (0.18, 0.86)*
Bullied outside school				
No	729 (18.3)	353 (71.8)	Ref	Ref
Yes	141 (29.8)	52 (59.1)	0.57 (0.37, 0.87)*	0.60 (0.20, 1.84)
Cyberbullied				
No	676 (17.69)	322 (72.80)	Ref	Ref
Yes	192 (30.18)	78 (57.97)	0.52 (0.31, 0.85)*	0.61 (0.26, 1.43)

^a^ AOR: Adjusted odds ratio, adjusted for age, suicide attempt, being physically attached, currently using marijuana, tried alcohol before age 14, tried cigarette before 14. * p-value < 0.05. **p-value < 0.001

### The association between bullying experiences and condom use

As shown in Table 4, adolescents who reported experiencing bullying at school had a significantly lower rate of condom use in their last sexual encounter compared to those who were not bullied at school (49.9% vs. 74.2%). The odds of using a condom in the last sexual encounter were significantly lower for those bullied at school than for those who were not (OR = 0.35, 95% CI: 0.19–0.64), and this association remained statistically significant after adjusting for potential confounders (AOR = 0.39, 95% CI: 0.18–0.86). Conversely, while adolescents who reported being bullied outside school also showed a lower rate of condom use in last sex compared to those not bullied outside school (59.1% vs. 71.8%), the association was significant only in unadjusted analysis (OR = 0.57, 95% CI: 0.37–0.87) and became non-significant after adjustment (AOR = 0.60, 95% CI: 0.20–1.84). Similar patterns were observed with cyberbullying: while the odds of condom use were lower among cyberbullied adolescents (57.97% vs. 72.80%; OR = 0.52, 95% CI: 0.31–0.85), this association was no longer significant in adjusted models (AOR = 0.61, 95% CI: 0.26–1.43).

## Discussion

Although the study primarily focused on the association between bullying victimization and condom use, it also shed light on potential psychosocial factors and substance use that correlate with condom use among school-going Thai students, offering valuable insights for public health interventions targeting adolescent sexual health in Thailand. One notable finding was the significant association between age and condom use, with older adolescents (> 14 years) being more likely to utilize condoms during sexual encounters compared to their younger counterparts. In addition, although insignificant, the rate of condom use among females was slightly lower among females (66.86%) compared to males (70.77%) which has significant implications for current sex education at schools. Thailand formally introduced sex education into its compulsory education curriculum in 2001, shifting the responsibility from homes to schools due to societal taboos [13]. Sex education is often combined with health education in Thai schools, typically starting in the third grade, when children are around 8 to 9 years old [14]. However, despite the efforts, there are concerns about the adequacy and comprehensiveness of sex education in Thai schools. These include outdated and age-inappropriate curricula, lack of emphasis on gender equality and sexual rights, and insufficient teacher training [14], which contributes to various issues, including misinformation, lack of awareness, and increased incidents related to sexual health [15]. In line with previous reports [16–18], the findings of the present study also highlight the importance of an age-appropriate sexual education tailored to adolescents at different developmental stages. It is also crucial to address gender norms and power dynamics in sexual relationships in sex education curricula to ensure equitable access to and utilization of preventive measures such as condoms among Thai adolescents [19–21].

Furthermore, the association between psychosocial factors and condom use revealed intriguing patterns. Despite the documented link between feelings of loneliness or experiencing anxiety with risky sex behaviors [22, 23], adolescents reporting these symptoms in our study did not show significant differences in condom use compared to their counterparts. Cultural factors such as stigma surrounding mental health disorders and societal taboos around sex as well as the sampling limitations such as the exclusion of out-of-school adolescents who exhibit higher depressive symptoms may contribute to divergent results. Despite that, in line with previous studies [24–26], adolescents reporting extreme psychological issues such as suicidal ideation and suicide attempts as well as those exhibiting interpersonal violence such as physical violence, or engagement in physical fights were significantly less likely to use condoms, which reinforce the potential impact of mental health in addition to interpersonal violence on sexual risk behaviors among Thai adolescents. On the other hand, social support emerged as a protective factor, with adolescents reporting close friendships demonstrating higher rates of condom use. It has been shown that supportive friendships can moderate the impact of stressful life events on sexual risk-taking [27]. However, the role of social support is complex, as it can also act as a stressor [28]. Nonetheless, this finding underscores the importance of fostering supportive social networks among adolescents to promote positive sexual health outcomes.

Another notable finding in our study was lower odds of condom use among those with current substance use, particularly marijuana and amphetamine/methamphetamine. This has been attributed to impaired judgment and decision-making regarding safe sex practices under the influence of drugs [29, 30]. Surprisingly, no significant association was found between current alcohol use and condom use during the last sexual encounter. This contradicts the previous studies that have found a relationship between alcohol consumption and risky sexual behaviors [31–33] and might be due to previously mentioned reasons such as sampling limitations and exclusion of out-of-school students as well as stigma around the drinking and having sex under the influence of alcohol which warrant further research. Despite that, initiating alcohol consumption and cigarette smoking before the age of 14 were associated with decreased odds of condom use during the last sexual encounter. This might be due to the higher risk-taking behaviors among those who initiate drinking and smoking at younger ages [34]. Early initiation of substance use may also lead to impaired decision-making abilities and struggling to make responsible choices regarding sexual health practices, such as condom use [33]. Further research is needed to explore this relationship more comprehensively and understand the nuances involved. Nonetheless, this underscores the importance of early intervention and prevention efforts targeting substance use initiation in adolescence to promote safer sexual practices.

Lastly, the most critical finding of this study is the significant association between bullying victimization at school and condom use, which has important implications for targeted intervention strategies. Adolescents who experienced bullying at school were significantly less likely to use a condom during their last sexual encounter, even after adjusting for confounding factors. This effect was not observed for bullying outside school or cyberbullying in the adjusted models, suggesting that the structured and peer-intensive school environment may amplify the psychological impact of bullying, leading to increased risk-taking behaviors such as unprotected sex. Consistent with previous research, these findings underscore the heightened vulnerability of adolescents who face bullying within school settings to engage in risky sexual health behaviors [1, 2, 6]. The stronger, adjusted association between bullying victimization at school and condom use during the last sex, compared to bullying outside of school, likely reflects the distinct psychological and social impacts of bullying within the structured school environment. Bullying at school occurs within a consistent peer setting, where daily interactions can intensify stress, reduce self-esteem, and heighten vulnerability to peer pressures, all of which are linked to increased risk-taking behaviors, including inconsistent condom use [6]. In contrast, bullying outside school may occur less frequently or across varied settings, resulting in less cumulative psychological impact on decision-making related to sexual health [35]. Moreover, in addition to condom use, studies have also indicated that adolescents who report bullying victimization fail to use other contraceptives, underscoring the complex relationship between sexual behaviors and experiences of bullying. The attenuation of the association between bullying outside school and cyberbullying with condom use in the adjusted model, however, warrants further research to elucidate the underlying mechanisms through which different types of bullying might affect mental health and consequent risky behaviors among adolescents.

Given these findings, intervention strategies should comprehensively address bullying in all its forms, with particular attention to school-based bullying. Anti-bullying programs in schools should promote a positive, inclusive culture and educate students on respectful relationships and conflict resolution. Mental health support should be accessible to students who have experienced bullying to help mitigate the emotional impact of victimization [36]. Additionally, mental health support services should be readily available for students who have experienced bullying, addressing the emotional and psychological effects of victimization [2]. To enhance condom use behaviors specifically, comprehensive sexual health education programs should be integrated into school curricula, emphasizing the importance of safe sex practices, including condom use. Furthermore, peer support groups or mentoring programs could be established to provide adolescents with a supportive network to navigate the challenges associated with bullying and promote healthy sexual decision-making. Lastly, collaboration between schools, parents, healthcare providers, and community organizations is essential to ensure a holistic approach to addressing bullying and promoting protected sexual behaviors among adolescents [2].

## Limitation

While this study offers valuable insights into the factors associated with condom use among Thai adolescents, it is important to consider several limitations. Firstly, relying on self-reported data could introduce biases such as recall and social desirability bias, potentially affecting the accuracy of responses, especially on sensitive topics like sexual behavior and bullying experiences. Additionally, the use of a cross-sectional design limits our ability to establish causality and temporal relationships between variables, making it challenging to infer causal effects. Furthermore, focusing solely on school-going adolescents may overlook the experiences of out-of-school youth, who may face different challenges related to sexual health. Moreover, the absence of data on certain psychosocial factors, including cultural norms, societal values, and the total population from which the sample was drawn, limits the scope of the analysis. Additionally, the lack of detailed information on the profiles of sex partners and other key contextual factors might have overlooked significant determinants of condom use among adolescents. Lastly, the generalizability of the findings may be restricted to the Thai context, cautioning against extrapolating results to other cultural or geographic settings.

## Conclusion

Although the study primarily focused on the association between bullying victimization and condom use, it also identified several other significant factors associated with condom use among Thai students. Notably, older age and being in higher grades were associated with increased condom use, highlighting the importance of tailoring sex education at Thai schools to different age groups. The findings also underscored the complex relationship between psychosocial factors, such as extreme psychological issues and experiences of interpersonal violence, and condom use behaviors necessitating appropriate interventions. Substance use, particularly marijuana and amphetamine/methamphetamine, emerged as significant barriers to condom use, emphasizing the importance of early intervention strategies targeting substance use initiation. Moreover, the detrimental impact of both traditional and cyberbullying on condom use underscores the critical role of intervention strategies aimed at bullying prevention.

## Data Availability

The GSHS 2021 is a publicly available dataset and is available on the World Health Organization NCD Microdata Repository website at: https://extranet.who.int/ncdsmicrodata/index.php/catalog/.
